# Evaluation of the Risk Factors for Retinopathy of Prematurity in Preterm Infants Admitted to the Neonatal Intensive Care Unit (NICU)

**DOI:** 10.7759/cureus.77163

**Published:** 2025-01-08

**Authors:** Sayash Arya, Vasu Saini, Sangram M. Bhosale, Shafeeque Kuniabdullah, Sana Irfan Khan

**Affiliations:** 1 Pediatrics, Shri Guru Ram Rai Institute of Medical and Health Sciences, Dehradun, IND; 2 Pediatrics, Government Medical College and Hospital, Chandrapur, IND; 3 Pediatrics, Boston Children's Hospital, Boston, USA; 4 Pediatrics, Flushing Hospital Medical Center, New York, USA

**Keywords:** apnea of prematurity (aop), blindness prevention, infant low birth weight, low apgar score, low birth weight infants, oxygen therapy, preterm, preterm babies, preterm birth, rop-retinopathy of prematurity

## Abstract

Background: Retinopathy of prematurity (ROP) is a disordered retinal blood vessel growth in preterm infants sustained in neonatal intensive care units (NICUs).

Objective: The objective of this study is to identify the risk factors linked to the occurrence of ROP in preterm infants.

Methods: This study was a prospective longitudinal study involving neonates hospitalized in the NICU at Shri Mahant Indiresh Hospital, Dehradun, India, from December 2018 to December 2019. A total of 320 preterm neonates were admitted, with only 113 included in our study due to the absence of parental permission, patients leaving against medical advice, or failure to attend follow-up appointments.

Results: In our study, 51 (45.13%) newborns developed ROP, while 62 (54.87%) did not, resulting in an incidence of 45.13%. Among 37 children diagnosed with hyaline membrane disease (HMD), 25 (67.57%) tested positive for ROP. Fifty percent of infants with an appearance, pulse, grimace, activity, and respiration(APGAR) score of 6 or lower at one minute had retinopathy, in contrast to just 32.69% of those with a score greater than 6. Anemia and the transfusion of blood products emerged as significant risk factors for the onset of retinal detachment. Meningitis was also identified as a significant risk factor for the development of retinopathy.

Conclusion: The findings indicate that low gestational age, low birth weight, HMD, oxygen therapy, and acute ophthalmopathy are independent risk factors associated with ROP development. Additional risk factors such as low APGAR score, anemia, surfactant therapy, abnormal cranial ultrasound, and blood product transfusion should be considered when monitoring preterm babies.

## Introduction

Retinopathy of prematurity (ROP) is a disordered retinal blood vessel growth in preterm infants sustained in neonatal intensive care units (NICUs) [1-3]. This condition might cause blindness or vision impairment in the absence of appropriate diagnosis and treatment. The most common cause of avoidable blindness among children is ROP [4].

The stages of ROP delineate the ophthalmoscopic observations at the interface of the vascularized and avascular retina: stage 1 features a subtle demarcation line, stage 2 presents an elevated ridge, stage 3 exhibits extraretinal fibrovascular tissue, stage 4 indicates subtotal retinal detachment, and stage 5 signifies total retinal detachment [5]. Furthermore, plus disease, characterized by considerable vascular dilatation and tortuosity in the posterior retinal arteries, can manifest at any stage and signifies augmented blood flow within the retina [6].

Prematurity is the primary risk factor for ROP. The incidence of ROP escalates with diminishing gestational age and birth weight. Nonetheless, not all preterm infants exhibit the development of ROP [5]. Significant risk factors that elevate the likelihood of developing ROP include oxygen therapy, anemia requiring blood transfusion, and apnea. Nonetheless, a very preterm, extremely low birth weight neonate may develop ROP even in the absence of oxygen exposure or other risk factors [4,5]. The All India Institute of Medical Sciences (AIIMS) protocol and the American Academy of Pediatrics (AAP) recommend screening for infants with a birth weight of less than 500 grams and a gestational age of less than 32 weeks. However, the National Neonatology Forum (NNF) proposes a threshold of less than 1750 grams in birth weight and less than 35 weeks in gestational age. In India, ROP has been seen in larger infants with a birth weight of 1500-2000 grams. The objective of this study is to discover the risk factors linked to the occurrence of ROP in all preterm newborns.

## Materials and methods

Study design, study period, and study participants

This study was a prospective longitudinal study involving neonates hospitalized in the NICU of Shri Mahant Indiresh Hospital, Dehradun, India, from December 2018 to December 2019. The Shri Guru Ram Rai Institute of Medical and Health Sciences Institutional Ethics Committee approved the study (approval number: SGRR/IEC/55/19). Our research included all preterm neonates with a gestational age of less than 36 weeks and a birth weight of less than 1750 grams who underwent an ophthalmologic examination for ROP screening.

Study procedure and data collection

During the period of one year, 320 premature neonates were brought to the NICU, of which only 113 were included due to the absence of parental permission, patients leaving against medical advice, or failure to attend follow-up appointments. Neonates were assessed in the newborn unit while maintaining asepsis under the supervision of the attending pediatrician. After screening for ROP, preterm infants were labeled clinically according to the International Classification of ROP (ICROP).

Inclusion Criteria

The inclusion criteria for this study encompassed preterm infants with a gestational age of 26 weeks or less and a birth weight of 1750 grams or below.

Exclusion Criteria

The exclusion criteria for this study included preterm infants with congenital anomalies or inborn errors of metabolism.

Time of Evaluation for ROP

The timing of evaluation in this study was based on gestational age. Preterm infants born at or before 30 weeks of gestation were evaluated at two weeks of life, while those born at or after 31 weeks of gestation were assessed at four weeks of life.

Neonates were assessed in the newborn unit while maintaining asepsis under the supervision of the attending pediatrician. Pupils were dilated using phenylephrine 2.5% and tropicamide 0.5%. Tropicamide was administered in one drop every 10-15 minutes, totaling four applications, commencing one hour prior to the planned assessment. Subsequently, phenylephrine was administered, one drop prior to the assessment. Phenylephrine was available in a 10% concentration and was diluted fourfold before application. ROP screening was conducted using indirect ophthalmoscopy utilizing a 20D lens by a proficient ophthalmologist. Following the application of a topical anesthetic drop, proparacaine, a wire speculum is used to maintain the separation of the eyelids. The examination begins with the anterior segment of the eye to assess the tunica vasculosa lentis, pupillary dilatation, and clarity of the lens/media; this is succeeded by an evaluation of the posterior pole for plus disease; and finally, a systematic assessment of all clock hours of the peripheral retina is conducted. Ophthalmological notes were recorded following each ROP assessment, specifying the zone, stage, and extent in clock hours of any ROP, as well as the existence of any pre-plus or plus disease, and were maintained with the infant's medical record.

Type of study

This was a prospective longitudinal study conducted in the Neonatal Intensive Care Unit (NICU) of Shri Mahant Indiresh Hospital. All neonates admitted to the NICU were registered after obtaining informed consent from their parents or guardians. Data regarding age, sex, birth weight, and gestational age were collected.

Statistical analysis

The data was analyzed by using IBM SPSS Statistics for Windows, V. 20.0 (IBM Corp., Armonk, NY, USA). Parameters were recorded and collected, data was analyzed by using standard statistical methods, and demographic characteristics were compiled by using Student's t-test and the chi-squared test as appropriate. Statistical test is significant at p < 0.05 at a 5% level of significance.

## Results

Based on the demographic profile, our study showed significant results for all variables except gender and mode of delivery. Neonates admitted with the following variables demonstrated significant findings.

Among the study population, a lower gestational age and birth weight were significantly associated with the presence of ROP. The mean gestational age in ROP-positive cases was 31.73 ± 2.44 weeks, compared to 33.73 ± 2.10 weeks in ROP-negative cases. Similarly, the mean birth weight in ROP-positive cases was 1.41 ± 0.370 kg, whereas ROP-negative cases had a higher mean birth weight of 1.69 ± 0.367 kg. These findings highlight the importance of low gestational age and birth weight as key risk factors for the development of ROP.

A low appearance, pulse, grimace, activity, and respiration (APGAR) score (<6) and the presence of hyaline membrane disease (HMD) were significantly associated with an increased incidence of ROP, demonstrating statistically significant correlations. Furthermore, the development of apnea of prematurity (AOP) and the administration of surfactant therapy were also strongly linked to the occurrence of ROP. Prolonged oxygen therapy emerged as another statistically significant risk factor, further underscoring the multifactorial etiology of ROP in this vulnerable population (Table 1).

**Table 1:** Demographic profile and risk factors associated with ROP Student's t-test and the chi-squared test are applied for demographic variables. Statistical test is significant at p < 0.05 at a 5% level of significance. ROP: retinopathy of prematurity; APGAR: appearance, pulse, grimace, activity, and respiration; HMD: hyaline membrane disease; AOP: apnea of prematurity; USG: ultrasonography; NA: not applicable

Data/parameter	Categories of the parameters	ROP present (number)	ROP present (percentage)	ROP absent (number)	ROP absent (percentage)	P-value	Chi-squared value
Gender	Male	35	50.7	34	49.3	0.1364	2.22
	Female	16	36.36	28	63.64		
Mode of delivery	Vaginal	37	51.39	35	48.61	0.0786	3.13
	Cesarean	14	34	27	66		
Gestational age	Weeks	31.73 ± 2.44	NA	33.73 ± 2.10	NA	0.0001	NA
Birth weight	Kg	1.41 ± 0.370	NA	1.69 ± 0.367	NA	0.0001	NA
APGAR (one minute)	<6	20	60.60	13	39.4	0.0128	6.39
	>6	17	32.69	35	67.31		
HMD	Yes	25	67.57	12	32.14	0.0011	11.17
	No	26	34.21	50	65.71		
Oxygen support	<5 days	24	42.1	33	57.9	0.0183	14.01
	5-10 days	15	93.75	1	6.25		
	>10 days	6	66.66	3	33.34		
AOP	Yes	22	78.57	6	21.43	0.0001	16.77
	No	29	34.12	56	65.88		
Surfactant	Yes	16	69.56	7	30.44	0.011	6.94
	No	35	38.88	55	61.12		
Cranial USG	Abnormal	14	66.66	7	33.34	0.0324	4.82
	Normal	37	40.22	55	59.78		

In our study, antenatal risk factors such as anemia and blood product transfusion were found to be significantly associated with the development of ROP. In contrast, antenatal steroid use and antenatal hypertension did not show statistically significant results in relation to ROP (Table 2).

**Table 2:** Relationship between blood analysis and antenatal history and ROP The chi-squared test is used for analyzing and presenting the data. A p-value of <0.05 (e.g., anemia and blood product transfusion) indicates a statistically significant association. A p-value of >0.05 (e.g., antenatal steroids and antenatal hypertension) suggests no statistically significant association in this dataset. ROP: retinopathy of prematurity

Parameter	Presence/absence of parameter	ROP present (number)	ROP present (percentage)	ROP absent (number)	ROP absent (percentage)	P-value
Anemia	Yes	13	72.22	5	27.78	0.0163
	No	38	40	57	60	
Blood product transfusion	Yes	13	68.42	6	31.58	0.0304
	No	38	4.42	56	95.58	
Antenatal steroid	Yes	20	54.05	17	45.95	0.1854
	No	31	40.79	45	59.21	
Antenatal hypertension	Yes	6	37.5	10	62.5	0.671
	No	45	46.39	52	51.61	

In this study, patients treated for meningitis showed statistically significant results for ROP, with a p-value of 0.01434. However, other complications such as neonatal jaundice (NNJ), sepsis, and seizures did not show statistically significant results, with p-values greater than 0.05 (Table 3).

**Table 3:** Relationship between complications and ROP The chi-squared test is used for analyzing and presenting the data. A p-value of <0.05 (e.g., meningitis; p = 0.0143) indicates a statistically significant association between meningitis and ROP. A p-value of >0.05 (e.g., NNJ, seizures, and sepsis) suggests no statistically significant association between these complications and ROP. ROP: retinopathy of prematurity; NNJ: neonatal jaundice

Complication	Presence/absence of parameter	ROP present (number)	ROP present (percentage)	ROP absent (number)	ROP absent (percentage)	P-value
NNJ	Yes	24	48	26	52	0.5855
	No	27	42.86	36	57.15	
Seizure	Yes	13	65	7	35	0.0544
	No	38	40.86	55	59.14	
Sepsis	Yes	38	47.5	42	52.95	0.4319
	No	13	39.39	20	60.61	
Meningitis	Yes	10	76.92	3	23.08	0.014344
	No	41	41	59	59	

## Discussion

Deficient vascular development in the retinal vascular systems of preterm newborns leads to ROP, resulting in sequelae and visual abnormalities, including retinal detachment, amblyopia, myopia, and other visual complications [7,8]. Nearly all preterm neonates with a history of NICU admission are at risk for this condition and require screening or treatment [9]. Although the survival rate of preterm neonates in NICUs globally has improved, the morbidities associated with prematurity are on the rise. ROP is a critical issue in these patients [10]. In our study of 113 neonates, 51 developed ROP, while 62 did not, resulting in an incidence of 45.13%. This finding is comparable to a study conducted by Adio et al. [11] in Nigeria in 2014. Consistent with our findings, a study conducted by Rasoulinejad and Montazeri [12] in 2016 revealed a prevalence of ROP at 45% among 306 neonates in Babol, Iran.

Rasoulinejad and Montazeri [12] identified prematurity, low birth weight, and oxygen therapy beyond five days as the most prevalent risk factors. Our research indicates that low birth weight and younger gestational age significantly impact the incidence of ROP. The overall mean gestational age was 31 weeks, with 81.8% at or below 28 weeks, 72.7% from 29 to 31 weeks, 63.6% in the 32-34-week range, and only 9% in the group with a gestational age at or below 35 weeks, consistent with studies such as Chawla et al. [13] conducted in New Delhi on the risk factors for ROP. HMD is another notable risk factor for ROP, with a p-value of 0.011 in our study. Among 37 children diagnosed with HMD, 25 (67.57%) tested positive for ROP, aligning with the cohort research of Kim et al. [14] including preterm newborns on ROP risk factors (HMD (p = 0.02)). This study reported that ROP is markedly more severe in infants with HMD.

The duration of supplementary oxygen is a critical factor contributing to ROP, with a p-value of 0.0183 in our study. Other studies identifying oxygen support as a significant risk factor for the development of ROP include Adio et al. in Nigeria, Kim et al., and Sears et al. (oxygen (p = 0.01)) [11,14,15]. The APGAR score at one minute was identified as a significant risk factor for the development of ROP in this investigation (p < 0.01). Fifty percent of infants with an APGAR score of 6 or lower at one minute had ROP, in contrast to just 32.69% of those with an APGAR score greater than 6. Additional studies that correlate with our findings include those by Freitas et al. [16], as well as Hu et al. in 2023 in China [17]. Anemia and the transfusion of blood products emerged as significant risk factors for ROP in our analysis, with p-values of 0.0163 and 0.0304, respectively. Seventy-two percent of newborns with anemia developed ROP, in contrast to just 40% of those without anemia. In a similar vein, 68.42% of infants who received any blood component had ROP, in contrast to merely 4.42% of those who did not receive any blood component. Additional research corroborating our findings include Freitas et al. [16] (p = <0.001 for both anemia and blood product transfusion), Arora et al. [18], and Adio et al. in Nigeria [11].

Our investigation revealed that meningitis is a significant risk factor for the development of ROP, with a p-value of 0.0143. Our findings are also corroborated by another investigation conducted by Kumar et al. (p < 0.05) [19]. In the current investigation, whereas seizures, sepsis, and NNJ were not identified as major risk factors for the development of ROP, they exhibited a high association with ROP, as the odds ratio exceeds 1. Certain investigations, such as the one by Freitas et al. [16], do not fully concur with our findings, as they identified statistically significant associations between seizures, sepsis, and ROP. The study supporting our findings, with p = 0.740, was performed by Alajbegovic-Halimic et al. [20].

The method of delivery has been shown to influence the occurrence of ROP. In this context, a study examining the mode of delivery and ROP by Manzoni et al. [21] noted the severe form of ROP among neonates born via vaginal delivery. AOP, with a p-value of 0.0001, is identified as an independent risk factor in our study. A total of 28 newborns experienced apnea, of which 22 (78.57%) subsequently developed ROP, in contrast to only 34.12% in the non-AOP group. It is comparable to the study conducted by Kim et al. and Freitas et al. [14,16].

Limitations

This study has some limitations. First, not all identified risk factors could be included. ROP is a multifactorial disease with numerous contributing factors, which introduces the possibility of selection bias in identifying potential risk factors. Second, the study was conducted in a single NICU. Although the NICU serves as a regional referral center where all admitted preterm infants receive standardized and advanced care, the sample size of preterm infants included in the study was relatively small. Despite these limitations, the study provides consistent conclusions and effectively distinguishes between the risk factors associated with the development and progression of ROP.

## Conclusions

The examination of risk factors for ROP development will facilitate comprehension and prediction in preterm infants. Timely retinal screening of high-risk preterm newborns is crucial to avert the progression of severe ROP. Given that ROP can result in severe consequences, including total blindness, it is imperative to implement measures to avert the progression of advanced ROP by enhancing newborn care and improving the identification of critical ROP signs.

The prevalence of ROP in this study was 45.13%. The findings indicate that low gestational age, low birth weight, HMD, oxygen therapy, and AOP are independent risk factors for the development of ROP. Additional risk factors such as low APGAR score, anemia, surfactant medication, abnormal cranial ultrasound, and blood product transfusion should be considered when monitoring preterm infants. In conclusion, our study was performed in a NICU center and illustrates the distribution within a single regional facility. This study offers a coherent conclusion and distinctly differentiates the risk factors associated with the onset and advancement of ROP.
